# Generation of multi-solitons and noise-like pulses in a high-powered thulium-doped all-fiber ring oscillator

**DOI:** 10.1038/s41598-019-54563-7

**Published:** 2019-12-04

**Authors:** Vasilii Voropaev, Aleksandr Donodin, Andrei Voronets, Dmitrii Vlasov, Vladimir Lazarev, Mikhail Tarabrin, Alexander Krylov

**Affiliations:** 1 Bauman Moscow State Technical University, Science and Education Center for Photonics and IR-Technology, Moscow, 105005 Russia; 2 P. N. Lebedev Physical Institute of the Russian Academy of Sciences, Frequency standards Laboratory, Moscow, 119991 Russia; 3 Fiber Optics Research Center of the Russian Academy of Sciences, Hollow-core fiber department, Moscow, 119333 Russia

**Keywords:** Mode-locked lasers, Fibre lasers

## Abstract

We report a study on the switching of the generation regimes in a high-powered thulium-doped all-fiber ring oscillator that is passively mode-locked with nonlinear polarization evolution technique with different pumping rates and cavity dispersion values. In one experimental setup, switching was observed between the noise-like pulse and the multi-soliton (in the forms of soliton bunches and soliton rain) regimes by the adjustment of the intracavity polarization controllers. We attributed this to the crucial influence of the nonlinear polarization evolution strength determined by such key parameters as saturation (over-rotation) power, linear phase bias, and nonlinear losses on the pulse evolution and stability. So the soliton collapse effect (leading to noise-like pulse generation) or the peak power clamping effect (generating a bunch of loosely-bound solitons) may determine pulse dynamics. Both the spectrum bandwidth and coherence time were studied for noise-like pulses by varying the cavity length and pump power, as well as the duration of solitons composing bunches. As a result, both noise-like pulses (with spectrum as broad as 32 nm bandwidth) and multi-soliton formations (with individual pulse-widths ranging from 748 to 1273 fs with a cavity length increase from 12 to 53 m) with up to 730 mW average power were generated at a wavelength of around 1.9 μm. The results are important for the realization of the broadband and smooth supercontinuum which can be used as a source for mid-IR vibrational spectroscopy of gas samples for breath analysis and environmental sensing.

## Introduction

Thulium-doped fiber (TDF) mode-locked lasers are highly promising for spectroscopic and sensing applications, including gas-tracing, light detection and ranging systems (LIDARs), seed sources for mid-IR optical parametric oscillators (OPOs) and supercontinuum generation, biomedical applications, optical communications, micromachining and defense^1^. To date, the generation of conservative^2–4^, dispersion-managed^5,6^ and dissipative solitons^7,8^ and similaritons^9^ has been realized in TDF lasers using various mode-locking techniques, including Kerr-nonlinearity-based nonlinear polarization evolution(NPE)^4–6,8–10^, nonlinear loop mirrors^11,12^, various saturable absorbers (SESAM^7,13^ carbon nanotubes^2,14,15^, graphene^16,17^, and other 2D structures^3,18^) and hybrid techniques^19,20^. This provides great flexibility in the configurations of lasers and to the variety of ultra-short pulse characteristics available.

Apart from regular single-pulse generation at a fundamental repetition frequency, much attention has been paid to different multi-pulse generation regimes of mode-locked fiber lasers: e.g., regular (bound solitons^21,22^) and irregular (noise-like pulse (NLP)^4,8,12^, soliton bunches^23^, soliton rain^24^, and rogue waves^25^). These are attractive not only as a platform for the fundamental study of the peculiarities of ultra-short pulse generation in fiber lasers but also for promising applications in optical fiber communications^26^ and sensing^27^. Due to their smooth spectrum averaged over an ensemble of numerous individual ultra-short sub-pulses with randomly varying amplitudes, widths, and distances between them, NLPs are highly promising for sensing applications based on low-coherence spectral interferometry^27^, including high-resolution interrogation of the Bragg gratings array^27,28^, data storage^29^, temperature profile measurements^30^, optical coherence tomography, and even gyroscopy^31^. Moreover, owing to their stochastic nature, NLPs are strictly beneficial for flat supercontinuum generation^32^ and micromachining^33^, since even high-energy NLPs propagate along rather long fiber segments without distortions, caused by nonlinearity and dispersion, suffered by regular pulses.

By now, NLPs have been generated in TDF lasers mode-locked with various techniques including nonlinear polarization evolution^4,8^, SESAM^34^, carbon nanotubes^35^, graphene^36^, and nonlinear loop mirrors^12,37^. The highest NLP spectrum width (approximately 300 nm) has been realized in an NPE mode-locked TDF laser with normal cavity dispersion^8^, whereas the highest NLP energy (250 nJ at 476 mW average power) has been obtained in a NPE mode-locked TDF laser with an anomalous dispersion cavity and with 45° tilted Bragg grating used as a polarizer^12^. Meanwhile, as it has been observed, the NLP generation regime usually follows by corresponding regular pulse generation with pump power growth, regardless of the total cavity group delay dispersion (GDD) sign and value when fast mode-locking mechanisms based on Kerr nonlinearity are used^4,8,12^. However, switching between different multi-pulse generation regimes in TDF lasers mode-locked by nonlinear polarization evolution at high pumping strength has not been widely investigated and realized, despite the flexibility of the NPE technique enabled by controlling its key parameters with appropriate adjustments to the intracavity polarization controllers, pumping rate, and cavity length. The NPE strength determined by such key parameters as saturation power (over-rotation), linear phase bias (set by cavity birefringence adjustment), and nonlinear losses, was numerically shown to be mainly accounted for corresponding multi-pulse generation regime attainment, such as soliton collapse leading to NLP generation or the peak power clamping effect bringing in multi-soliton emissions^38–41^.

Moreover, apart from influence of the mode-locking mechanism, multi-soliton generation is strongly affected by different interactions between solitons, dispersive waves, and the continuous-wave (CW) noisy background circulating in the cavity—as well as nonlinearity and dispersion, gain relaxation dynamics, electrostriction, and acoustic effects^42,43^. Depending on the contribution from each of these types of interactions, various multiple states can be realized, including tightly- and loosely-bound solitons, soliton rain, and bunches of irregularly moving solitons^41–43^. Thus, due to their prospective fundamental and practical applications, multi-pulse state generation dynamics and switching should be studied experimentally while improving the appropriate laser characteristics.

In this work we demonstrate and study the switching of generation regimes in a high-powered thulium-doped all-fiber ring oscillator that is passively mode-locked with a nonlinear polarization evolution technique with different pumping rates and cavity dispersion values. We observed switching between the NLPs and the the multi-soliton (in the form of soliton bunches and soliton rain) regimes by adjustment of the intracavity polarization controllers. Spectrum bandwidth and coherence time of NLPs as well as pulse duration and bandwidth of multi-soliton regimes were studied depending on cavity length and pump power. As a result, both NLPs (as broad as 32 nm bandwidth) and multi-solitons (with individual pulse-widths ranging from 748 fs to 1273 fs with a cavity length increase from 12.8 m to 53 m) with up to 730 mW average power were generated at a wavelength of around 1.9 μm. The remainder of this paper is organized as follows: Section 2 describes the main optical components of the laser and measurement devices used in this work. Section 3 gives the description of three generation regimes achieved in the cavity, such as NLP, soliton bunches and soliton rain regimes. In Section 4 we discuss possibility of stable pulse generation in developed schemes by theoretical evaluation of single pulse generation and NPE mode-locking thresholds.

## Experimental Setup

**Figure 1:**
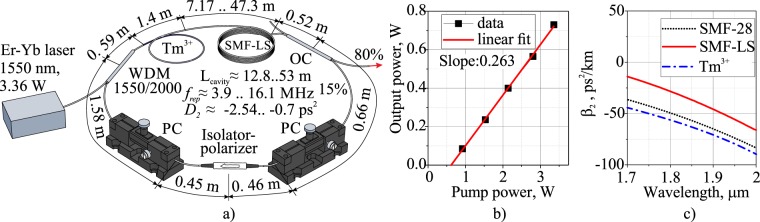
(**a**) Schematic of a thulium-doped fiber ring laser with NPE mode-locking, WDM - wavelength-division multiplexer, PC - polarization controller, Tm^3+^ - thulium-doped fiber, OC - output coupler; (**b**) Average output power dependence of TDF mode-locked laser on the pump power with a 10.16 m-long SMF-LS fiber inside; (**c**) Group velocity dispersion of cavity fibers.

The ring cavity of a TDF laser mode-locked by NPE technique is depicted in Fig. 1a. We used a home-made CW erbium/ytterbium-co-doped fiber laser with maximum power of 3.36 W operating at a wavelength of 1550 nm. The laser was used to pump a 1.4 m-long segment of a step-index (Δn = 0.012, core diameter *d*_*c*_ = 10 μm) thulium-doped aluminum-silica (0.8 wt% thulium, 3.6 wt% aluminum) glass fiber, with 44.5 dB/m small-signal absorption value at a wavelength of 1550 nm and anomalous group velocity dispersion (GVD) of ${\beta }_{2}=-\,70.8\,p{s}^{2}/km$ at a wavelength of 1900 nm. To couple pump radiation into the active fiber, we used a fused wavelength-division multiplexer WDMC-1x2-2000/1550 (Opneti Communications Co. Ltd, Hong Kong, China) based on single-mode fiber (SMF-28, Coning Inc., NY, USA).

We used an additional SMF-LS (Corning Inc., NY, USA) fiber with the GVD of ${\beta }_{2}=-\,45.8\,p{s}^{2}/km$ at a wavelength of 1900 nm to increase the intracavity nonlinearity and, as a result, reduce NPE mode-locking threshold, as shown in the discussion section. GVD of the SMF-LS fiber is less than that of the SMF-28 (Corning inc., NY, USA) fiber (−$64.96\,p{s}^{2}/km$). The length of the SMF-LS fiber was varied during the experiments from 7.17 to 47.3 m (the total cavity length ranged from ≈12.8 to 53 m), with corresponding intracavity GDD variation from −2.54 to −0.7 ps^2^. GVDs of fibers composing the cavity dependencies on the wavelength are depicted in Fig. 1c. We used a fused fiber coupler with internal losses of ≈5%, extracting 80% incident power from the cavity in order to enhance the efficiency of the TDF laser. The NPE mode-locking operation of the TDF laser was introduced by inserting a polarization-maintaining (PM) fiber pigtailed isolator-polarizer PMIS-S-2000-F-250 (Opneti Communications Co. Ltd, Hong Kong, China) with a blocked fast axis that also assured unidirectional generation in the cavity, and two squeezing-type polarization controllers (PCs) (PLC-006, General Photonics corp., Chino, USA) located near both ends of the PM-fiber. Figure 1b shows the dependence of the output average power of the TDF mode-locked laser on the pump power with a 10.16-m-long SMF-LS fiber inside. We measured average power at the laser output in pulsed regime as high as 730 mW with 26% slope efficiency and maximum pump power of 3.36 W. The TDF laser generation threshold was approximately measured to be 620 mW in terms of the pump power value. For different SMF-LS fiber lengths, the maximum output power varied slightly, but the mode-locking threshold changed significantly, this will be shown in the discussion section. Train of mode-locked pulses was registered with PD24-005-HS (IBSG Co., Ltd., Saint-Petersburg, Russia)^44^ photodiode with a bandwidth of 5 GHz, together with 1 GHz digital oscilloscope Wavesurfer104MXs-B (Teledyne LeCroy GmbH, Heidelberg, Germany) and high-speed oscilloscope MSOV334A (Keysight Technologies Ltd, Santa Rosa, USA) with a bandwidth of 33 GHz. The radio-frequency spectra were measured by means of an FSL (Rhode & Schwartz, Munich, Germany) spectrum analyzer. Optical spectra were recorded with both the MDR-206 monochromator (Lomo Photonics, Saint-Petersburg, Russia) with 300 lines/mm diffraction grating density and the PbSe Preamplified Detector PDA20H(-EC) (Thorlabs Inc., Newton, New Jersey, United States), along with an optical Fourier spectrometer, OSA 207C (Thorlabs Inc., Newton, New Jersey, United States). The autocorrelator (pulseCheck (APE Angewandte Physik & Elektronik GmbH, Berlin, Germany)) with non-collinear geometry, which is suitable for tight observations of the pulse pedestal in the scan range of 15 ps, was used to measure pulse width.

## Results

By random optimization of the PCs, a number of different multi-pulse generation regimes were achieved at various pumping strengths and cavity GDD. Among them, we identified three typical multi-pulse generation regimes observed in each laser scheme with different cavity lengths at high average power: viz., noise-like pulses, soliton rain, and bunches of loosely bound solitons. These regimes were obtained with different nonlinear cavity effects causing various mechanisms of pulse generation, such as soliton collapse for noise-like pulses and peak power clamping for soliton-like regimes. The main mechanism of separation of the generation regimes was the adjustment of PCs, which changed the evolution of polarization inside the cavity. All three generation regimes in the laser existed separately at the different angles and the compression ratio of fibers in the PCs, regardless of the pump power. The angle of rotation and the compression ratio of fibers in PCs for multi-solitons generation regimes were not differ much. Moreover, sometimes the soliton rain regime flowed into the soliton bunch generation regime without changing the settings of the PCs. Achieved generation regimes are described in details below.

### Noise-like pulses (NLPs)

**Figure 2:**
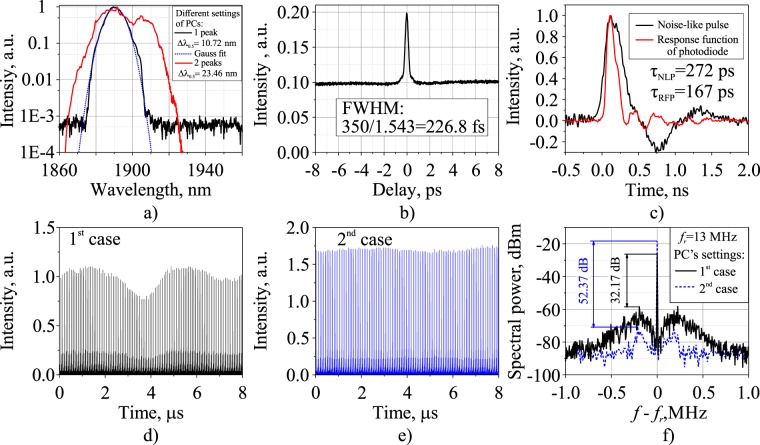
Characteristics of the noise-like pulse regime at 500 mW output laser power in the cavity with the 10.16 m-long SMF-LS fiber: (**a**) optical spectra, (**b**) intensity autocorrelation, (**c**) individual noise-like pulse with response function of photodiode (RFP), (**d**) pulse train at a fundamental repetition rate with enhanced amplitude modulation and (**e**) reduced amplitude modulation, and (**f**) radio frequency spectrum in the vicinity of the fundamental repetition frequency.

An NLP is a packet of a large number of ultra-short sub-pulses, with randomly varying amplitudes, durations, and delays between them^45^. An NLP has a typical duration of 10–100 ps and possesses low intrapulse and pulse-to-pulse coherence^46^ and a random intrapulse phase distribution^33^. It propagates along a cavity as a single unit at a fundamental repetition frequency, with its integral characteristics (the total bunch width and energy) fluctuating around their average values within a range from units to tens of percentages from one bunch to another^47^. It is noteworthy that NLPs have a typical two-scale autocorrelation trace of pulse intensity with a narrow coherent spike at zero delay located on the broad pedestal^32^. Figure 2 shows the basic integral characteristics of NLPs at 500 mW output power in the cavity with the 10.16 m-long SMF-LS fiber.

The main features of this generation regime are a broad smooth spectrum (Fig. 2a) and a significant pedestal inherent to intensity (Fig. 2b) autocorrelation trace. Meanwhile, the full width at half maximum (FWHM) of the coherence spike was measured to be 226 fs with a pedestal-to-peak ratio of ≈0.5, as retrieved from Fig. 2b. Due to the limited scan range of the autocorrelator, we were not able to measure the duration of the pedestal and observe the double-scale structure. The measured optical spectrum (Fig. 2a) of the NLPs generally has a Gaussian shape (blue dotted line), which is quite natural due to its averaging over numerous spectra of individual sub-pulses. However, in each scheme at some polarization controllers’ settings the NLP spectrum has a more complex shape with two peaks as seen in Fig. 2a (red curve). Figure 2c shows the waveform of an individual noise-like pulse along with the response function of the photodiode. Oscillograms have a tail that is associated with oscillations in the electrical circuit of the photodetector. It can be seen that the duration of the noise-like pulse (FWHM of 272 ps) is longer than the duration of the photodiode response function (FWHM of 167 ps) by 105 ps. However, it is impossible to accurately measure the duration of a noise-like pulse based on oscillograms; the estimated duration of NLP is about 100 ps.

Depending on the polarization controllers’ settings, NLPs emitted at a fundamental repetition frequency have different pulse-to-pulse energy fluctuations manifested as amplitude modulation of the pulse train, as seen in Fig. 2d,e. This peculiarity of the NLP generation regime^35^ appears as broad side-bands in the averaged RF spectrum, as depicted in Fig. 2f. Less stable NLPs have a reduced signal-to-noise ratio. Note that the amplitude modulation strength can be changed simply by appropriate PCs’ adjustment, although it is hardly possible to eliminate this modulation altogether.

**Figure 3:**
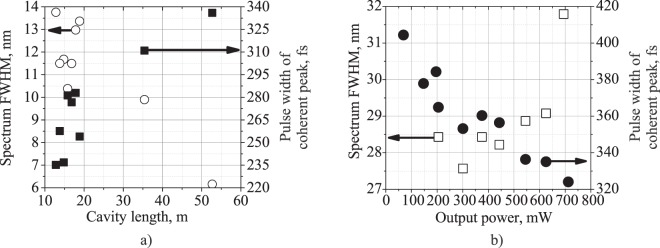
(**a**) Coherence spike width and Gaussian shape spectrum FWHM dependence on the cavity length at 500 mW output average power; (**b**) Coherence spike width together with complex spectrum FWHM dependence on the output average power of a TDF mode-locked laser with a cavity length of 55 m.

Figure 3a shows the coherent spike width and Gaussian shape spectrum FWHM evolution with a cavity length increasing at 500 mW output average power. Thus, as expected, the NLP coherence time generally increases from 235 to 336 fs and spectrum FWHM decreases from 13.76 nm to 6.16 nm with the cavity length (and total cavity GDD) increasing from 12.8 to 53 m. Figure 3b shows the dependence of coherent spike width and complex spectrum (with two peaks) FWHM evolution on the output average power (at a cavity length of 55 m) measured with the same PC settings. With output power increasing from 50 mW to 730 mW, the NLP coherence time goes down from 404 to 324 fs, while the spectrum width rises from 27.5 to 31.79 nm. When the laser operated at a maximum output power of 730 mW, the NLP repetition frequency doubled. With slight adjustments to the PCs, however, it was possible to achieve the fundamental repetition frequency again. Thus, taking into account the evolution of the coherence time and spectrum FWHM with both an increase to the cavity GDD and to the output average power (and energy) observed for an NLP pulse (Fig. 3), it is possible to ascribe some aspects of solitonic behavior to the sub-pulses constituting the NLP, despite their stochastic nature.

### Multiple soliton generation regimes

#### Soliton rain

**Figure 4:**
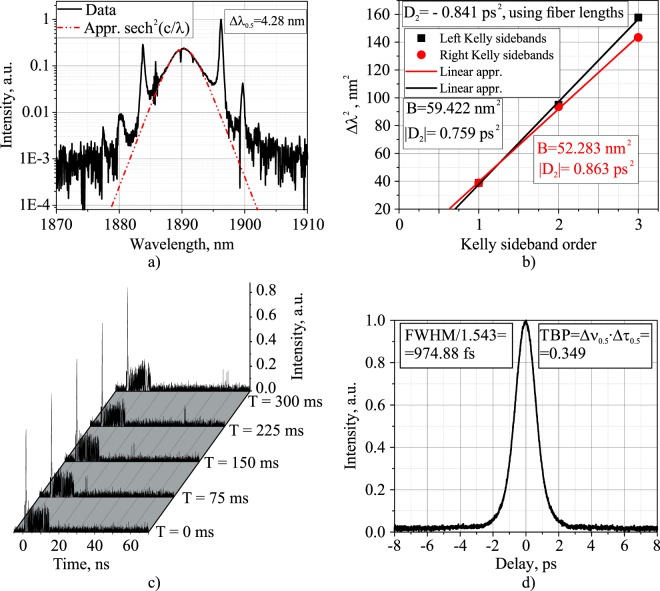
Characteristics of the soliton rain regime of operation in laser scheme with SMF-LS fiber of 10.16 m and output power of 500 mW: (**a**) optical spectrum, (**b**) squared Kelly side-peak position versus peak order for the left- and right-side peaks, (**c**) oscilloscope traces at different times, (**d**) intensity autocorrelation trace.

**Figure 5:**
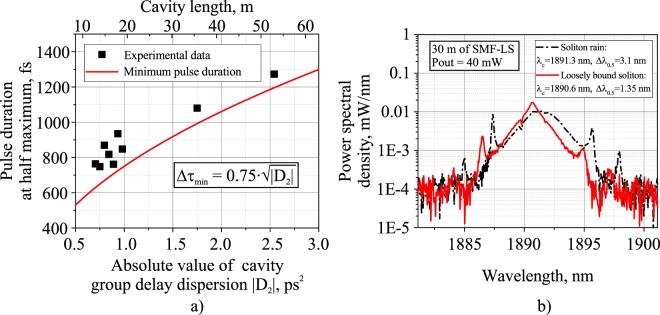
(**a**) Experimentally measured average duration of intra-bunch solitons at different cavity GDDs together with the theoretical dependence of the minimum soliton width; (**b**) optical spectra of the operation regimes of soliton rain and loosely bound soliton bunches of the TDF mode-locked laser with a 30 m SMF-LS fiber in the cavity and 40 mW output power.

The dynamics of soliton rains were first described in detail by Chouli and Grelu^24^. Currently, there are several theoretical and experimental studies on the dynamics of soliton rains in fiber lasers with various active fibers: viz., erbium-doped^43,48,49^, thulium-doped^50–52^, and ytterbium-doped^53–55^ fibers. Figure 4 shows the integral characteristics of a TDF mode-locked laser in soliton rain (SR) operation regime in the scheme with a 10.16-m-long SMF-LS fiber at a 500 mW output average power. The SR spectrum of 4.28 nm FWHM (Fig. 4a) is well approximated with an inherent to conservative soliton sech^2^-function, while displaying sharp high-amplitude Kelly side-peaks located symmetrically^56,57^. The dips in the spectra in Figs. 4a and 5b correspond to the air absorption lines, according to the HITRAN database^58^, because the radiation in the spectral measuring devices propagates in the air. On the other spectra presented in this article, these dips are not visible, because they were recorded using a monochromator, the resolution of which does not allow to resolve the dips. Taking into account the position of the Kelly peaks, we estimated the total GDD of the cavity using the following expression^59^:

1 $$|{D}_{2}|=\frac{{\lambda }_{s}^{4}}{\pi {c}^{2}B},$$

where *λ*_*s*_ is the central soliton wavelength, and *B* denotes the slope of linear dependence of the squared Kelly peak shift from the center wavelength (Δ*λ*^2^) on the Kelly sideband order, as shown in Fig. 4b. The absolute values of the total cavity GDD calculated on the basis of left- and right-side Kelly peaks are quite close to each other and amount to 0.759 ps^2^ and 0.863 ps^2^, respectively. Meanwhile, the cavity GDD value calculated using dispersions of fibers appears to be $|{D}_{2}|=0.841\,p{s}^{2}$, which is in a good agreement with previous estimations based on the Kelly peaks. Figure 4c shows the oscilloscope snapshots of SR regime at different instants in time in the same scheme with a 10.16 m-long SMF-LS fiber inside. Nonetheless, it is evident that solitons move from one condensed phase to another, 77 ns apart from each other (corresponding to the roundtrip time of a 16-m-long ring cavity). This is also an essential feature of SR regime^24,60^. Figure 4d shows the autocorrelation trace registered in SR regime at the same output power of 500 mW. The FWHM of the pulse duration is 974 fs, whereas the time-bandwidth product (TBP) is 0.349, which is close to the theoretical value inherent to conservative soliton (0.315). Moreover, the autocorrelation trace of the pulse intensity has a very low pedestal. This, along with sech^2^-shaped spectrum with pronounced Kelly peaks, is the evidence of the generation of almost identical solitons with equal durations and peak powers.

Another essential peculiarity inherent to soliton rain regime is the presence of a quasi-CW noisy background in the cavity. This forms from the generation of spurious CW and dispersive waves (continuously emitted during soliton propagation along the ring), and is a source of both new solitons via modulation instability and long-range interaction leading to their drift between condensed states^24,60^. This component corresponds to the effect of the pump power clamping when the excess power propagates as a CW radiation—and, upon reaching a critical level, converts to a new soliton or a bunch. Thus, in addition to the generation of strong Kelly peaks, a weak CW peak is evident at the short-wavelength wing of the SR spectrum, as shown in Fig. 4a. As the group velocity of the pulses which are generated at the wavelength of the CW component is higher than that of the solitons in the condensed phase (according to the dispersion of the cavity fibers), new solitons move faster than condensed states (Fig. 4c). At some particular PC settings, the condensed phase of solitons completely disappeared, resulting in solitons filling the entire cavity.

#### Soliton bunch generation at a fundamental repetition rate

**Figure 6:**
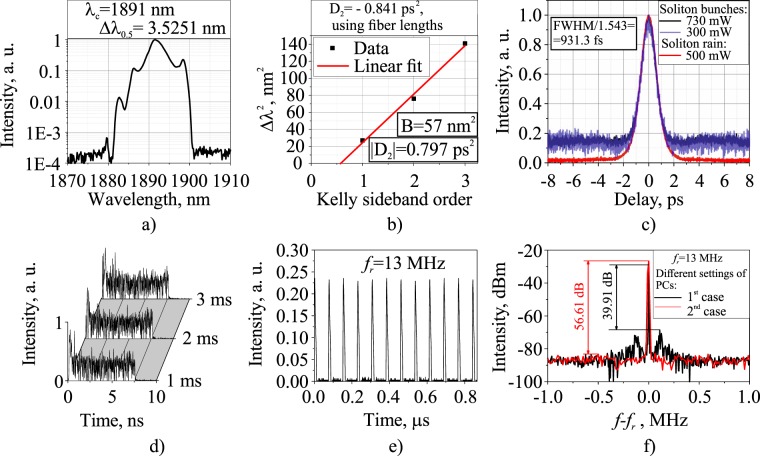
Characteristics of the loosely bound soliton bunch regime in laser scheme with a 10.16 m SMF-LS fiber and 500 mW output average power: (**a**) optical spectrum, (**b**) squared Kelly side-peak position versus peak order for the left-side peaks under the same conditions, (**c**) intensity autocorrelation traces of soliton rain regime at 500 mW output power (red curve) and loosely bound soliton bunch regime at 300 mW (blue curve) and 730 mW (black curve) output power, (**d**) soliton bunch captured on a high-speed oscilloscope at different time points, (**e**) pulse train, (**f**) radio-frequency spectrum in the vicinity of the fundamental frequency for different PCs’ settings.

Figure 6 shows the integral characteristics of soliton bunches propagating at a fundamental repetition frequency in laser scheme with 10.16-m-long SMF-LS in the cavity and average output power of 500 mW. It is noteworthy that a similar generation regime was interpreted as a bunch of irregularly oscillating solitons (loosely-bound solitons (LBSs)) in^61,62^. Figure 6a shows the optical spectrum with 3.52 nm FWHM. The shape of the spectrum is poorly approximated with the sech^2^-function, despite the presence of visible Kelly side-peaks sufficiently broader and weaker than those measured in soliton rain generation regime. Following the same procedure as that for soliton rain regime, we plotted the dependence of the squared Kelly peak spacing from the central wavelength on the peak order (as shown in Fig. 6b) to calculate GDD according to Eq. 1. Thus, the absolute value of the total GDD is 0.797 ps^2^, which is close to the cavity GDD calculated using the dispersions of fibers composing the ring ($|{D}_{2}|=0.841\,p{s}^{2}$). Figure 6c shows the intensity autocorrelation traces of loosely-bound solitons at average output power of 300 mW and 730 mW together with those of soliton rain regime (red curve) at 500 mW output power. The autocorrelation width is almost independent of the average power at the laser output, which serves as evidence that the peak power clamping effect (rather than soliton collapse) is responsible for the generation of both soliton rain and bunches of loosely bound soliton regimes. Indeed, since the soliton width and peak power are closely related according to the soliton area theorem, the peak power clamping effect limits the soliton peak power—and, of course, its duration—while increased pump power leads to the origination of new solitons through the pulse splitting effect or modulation instability of the dispersive wave field. The pedestal-to-peak ratio for an LBS autocorrelation trace was estimated to be ≈0.15, much large than that in the case of soliton rain regime. It was, in turn, ≈3.3 times lower than the pedestal-to-peak ratio for noise-like pulses, which is about 0.5. This fact, together with suppressed and broadened Kelly peaks, explains the propagation of a bunch of pulses with slightly different widths and peak powers and randomly varying distances between them^61,63^. Indeed, irregular soliton oscillations inside a bunch lead to the chaotization of dispersive waves (emitted by individual solitons) with noticeably deteriorating interaction coherence of the solitons and dispersive waves.

The pulse train of loosely bound soliton bunches circulating at the fundamental repetition frequency of 13 MHz is shown in Fig. 6e. There is some weak amplitude modulation of the pulse train related to bunch-to-bunch energy fluctuations, and this is also manifested as sidebands in the radio frequency (RF) spectrum plotted in Fig. 6f. The signal-to-noise ratio varies from 39.9 to 56.6 dB depending on the PCs’ settings. It is worth noting that RF spectra of both noise-like pulses and loosely-bound soliton bunches have characteristically broad side-bands near the fundamental frequency peak. This is further evidence in favor of increased deviation in the parameters (viz., the duration, peak power, and central wavelength) of intra-bunch solitons in comparison to soliton rain generation regime. The individual bunches of solitons observed with high-speed oscilloscope at different time points are depicted in Fig. 6d. Soliton distribution inside the bunch changes randomly over time what corresponds to soliton interactions with slightly different durations, peak powers and spectra in the group. However, the average bunch duration does not change in time and is approximately 7.5 ns. Figure 5a shows the experimentally measured average duration of intra-bunch solitons at different cavity GDDs together with the theoretical dependence of the shortest soliton duration limited by instabilities related to intense Kelly peaks generation from the coherent interaction between soliton and dispersive waves^64–66^. It is clearly seen that the pulse duration is close to this theoretical limit, although it slightly exceeds it for cavity GDD values. Since the soliton duration is unambiguously related to its peak power according to the soliton area theorem, we can suppose that the peak power clamping effect responsible for multi-soliton generation is, apart from NPE strength, closely related to soliton perturbations imposed by its interaction with dispersive waves. Moreover, we experimentally observed periodical switching between soliton rain regime and LBS bunch regime at particular PC settings and pumping strength. This switching was thereby induced by spurious perturbations to the laser, confirming the resemblance of the pulse generation dynamics of SR and LBS regimes. The average pulse spectra in these two regimes are shown in Fig. 5b indicating strictly different spectrum shapes inherent to the SR and LBS bunch regimes. Therefore, we expect that some subtle effects (such as electrostriction or acoustic effects) acting as perturbations of the laser cavity can also affect the long-range soliton interaction strength, thus leading to soliton bunching.

## Discussion

Finally, we characterize possible individual pulses composing soliton-like regimes in terms of energy, peak power and duration. Moreover, in order to examine a possibility of single soliton generation at the fundamental repetition frequency in the TDF laser, we compare mode-locking threshold power measured at different cavity lengths with output average power calculated for fundamental solitons. In this regard, we assume that solitons just before leaving the cavity (at the fiber coupler position) are fundamental ones, which is quite reasonable since propagation of amplified pulses in rather long SMF-LS passive fiber segments with anomalous GVD and low attenuation leads their shape to that of fundamental soliton. The energy, the peak power, and the average power of the solitons at the laser output (after coupler) are described by the following expressions^66^:

2 $${E}_{s}=K\frac{3.53|{\beta }_{2}|}{\gamma {\tau }_{s}},{P}_{s}=K\frac{3.11|{\beta }_{2}|}{\gamma {\tau }_{s}^{2}},{P}_{av}={f}_{rep}{E}_{s},$$

**Figure 7:**
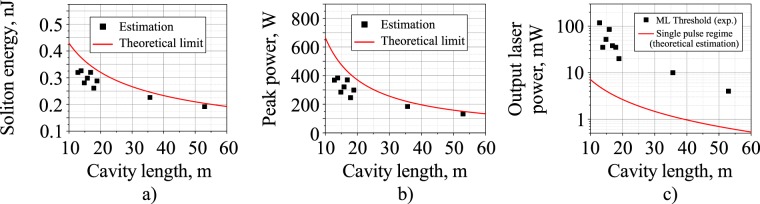
(**a**) Energy and (**b**) peak power of a single soliton at the laser output for the minimum possible pulse duration and for the durations obtained from the experiment, depending on the cavity length. (**c**) Average power of the fundamental soliton regime for the measured and minimum pulse durations and mode-locking threshold depending on cavity length.

Here *K* is the output coupling ratio (K = 0.8); nonlinearity coefficient (*γ*) and GVD of SMF-LS fiber (|*β*_2_|) at wavelength of 1900 nm are equal $0.56\,{W}^{-1}\,k{m}^{-1}$, $45.8\,p{s}^{2}/km$, respectively; ${\tau }_{s}$ is a soliton duration at half maximum; *f*_*rep*_ is a pulse repetition rate. Thus, the energy (Fig. 7a) and peak power (Fig. 7b) dependence on the cavity length at the laser output for minimum possible soliton duration (${\tau }_{s}=0.75\sqrt{|{D}_{2}|}$^66^) at given cavity GDD values are shown (red curve) with those calculated using measured values of ${\tau }_{s}$ for soliton-like regimes (black squares). Figure 7c demonstrates both experimentally observed average power (black squares) at the TDF laser output measured for mode-locking threshold condition and calculated average power (red curve) for fundamental soliton duration at different cavity lengths. As apparently seen in Fig. 7c mode-locking threshold power, apart from being strongly dependent on cavity length, substantially exceeds output average power in the case of nearly fundamental soliton generation regime, especially for less than 20 m-length cavities. It means that multi-soliton bunch emission occurs (with in excess of 10 solitons per bunch) even at mode-locking threshold for any cavity length from 13 m to 53 m. Moreover, output average power and energy calculated for single-soliton generation regime does not exceed 7 mW and 0.4 nJ respectively even at 13 m cavity length, gradually reducing with further cavity expansion. Nonetheless, mode-locking threshold power noticeably falls with cavity length increase which is inherent to NPE mode-locking mechanism due to evident enhancement of lumped nonlinearity governing polarization ellipse rotation in a cavity with extended length. Thus, according to our observations, there are two possible ways for single soliton generation to be realized in the developed scheme of the TDF mode-locked laser, which are both based on the mode-locking threshold reduction below fundamental soliton energy limit: (i) noticeable cavity elongation leading to inevitable decrease in soliton energy and power^12^ or (ii) implementation of a saturable absorber with reduced saturation power (and energy) such as carbon nanotubes or graphene instead of or together with NPE^2^. However, the first solution should have additional experimental confirmation.

## Conclusion

In this work, we demonstrated and studied the switching between three generation regimes in a high-powered thulium-doped all-fiber ring oscillator passively mode-locked with the NPE with respect to a 1.55 μm pumping rate and cavityGDD. We observed switching between the generation of NLP and the emission of multiple solitons (in the form of soliton bunch and soliton rain regimes) by adjusting the intracavity polarization controllers. Both the spectrum bandwidth and coherence time were studied for NLPs by varying the cavity length and pump power, as well as the duration and bandwidth of solitons. As a result, both NLPs (as broad as 32 nm bandwidth) and multi-solitons (with individual pulse-widths ranging from 748 to 1273 fs with a cavity length increasing from 13 to 53 m) with up to 730 mW average power were generated at a wavelength of around 1.9 μm. The results reported here could be important for the realization of the broadband and smooth supercontinuum^67^ which can be used as a source for mid-IR vibrational spectroscopy of gas samples for breath analysis and environmental sensing^1^. It can be conducted through development of a fiber amplifier for achieved pulses to significantly increase peak power and use output pulses to pump highly nonlinear fibers, such as chalcogenide nano-spiked waveguides^68^.
